# Supplementary material for: Causal interplay between lactose intolerance and gut microbiota: a combined bidirectional Mendelian randomization and in vivo validation study
Source: Front Nutr. 2026 Jun 1;13:1803337. doi: 10.3389/fnut.2026.1803337 (PMC13265576; doi:10.3389/fnut.2026.1803337)
Supplement: Supplementary file 1 [file Data_Sheet_1.zip › supplementary materials/Forward/leave one out cross validation/ebi-a-GCST90027625.finngen_R12_E4_LACTONAS.pdf]

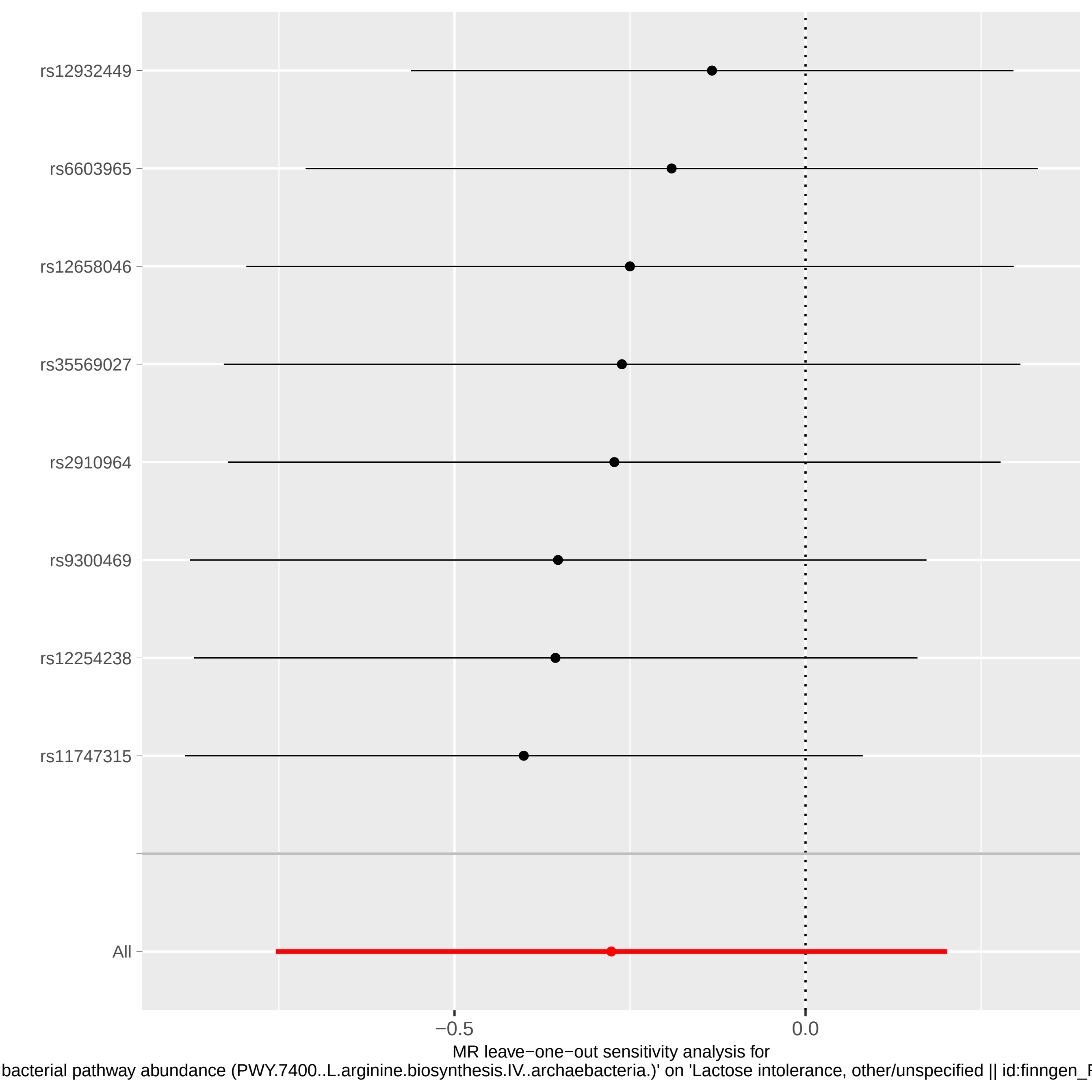

rs12932449
rs6603965
rs12658046
rs35569027
rs2910964
rs9300469
rs12254238
rs11747315
All
−0.5
0.0
MR leave−one−out sensitivity analysis for
bacterial pathway abundance (PWY.7400..L.arginine.biosynthesis.IV..archaebacteria.)' on 'Lactose intolerance, other/unspecified || id:finngen_F
